# Supplementary material for: Comparing the Fasting and Random-Fed Metabolome Response to an Oral Glucose Tolerance Test in Children and Adolescents: Implications of Sex, Obesity, and Insulin Resistance
Source: Nutrients. 2021 Sep 25;13(10):3365. doi: 10.3390/nu13103365 (PMC8538092; doi:10.3390/nu13103365)
Supplement: Supplementary file 1 [file nutrients-13-03365-s001.zip › nutrients-1305571-supplementary/Supplementary Files/Figure S1-S7.pptx]

## Slide 1
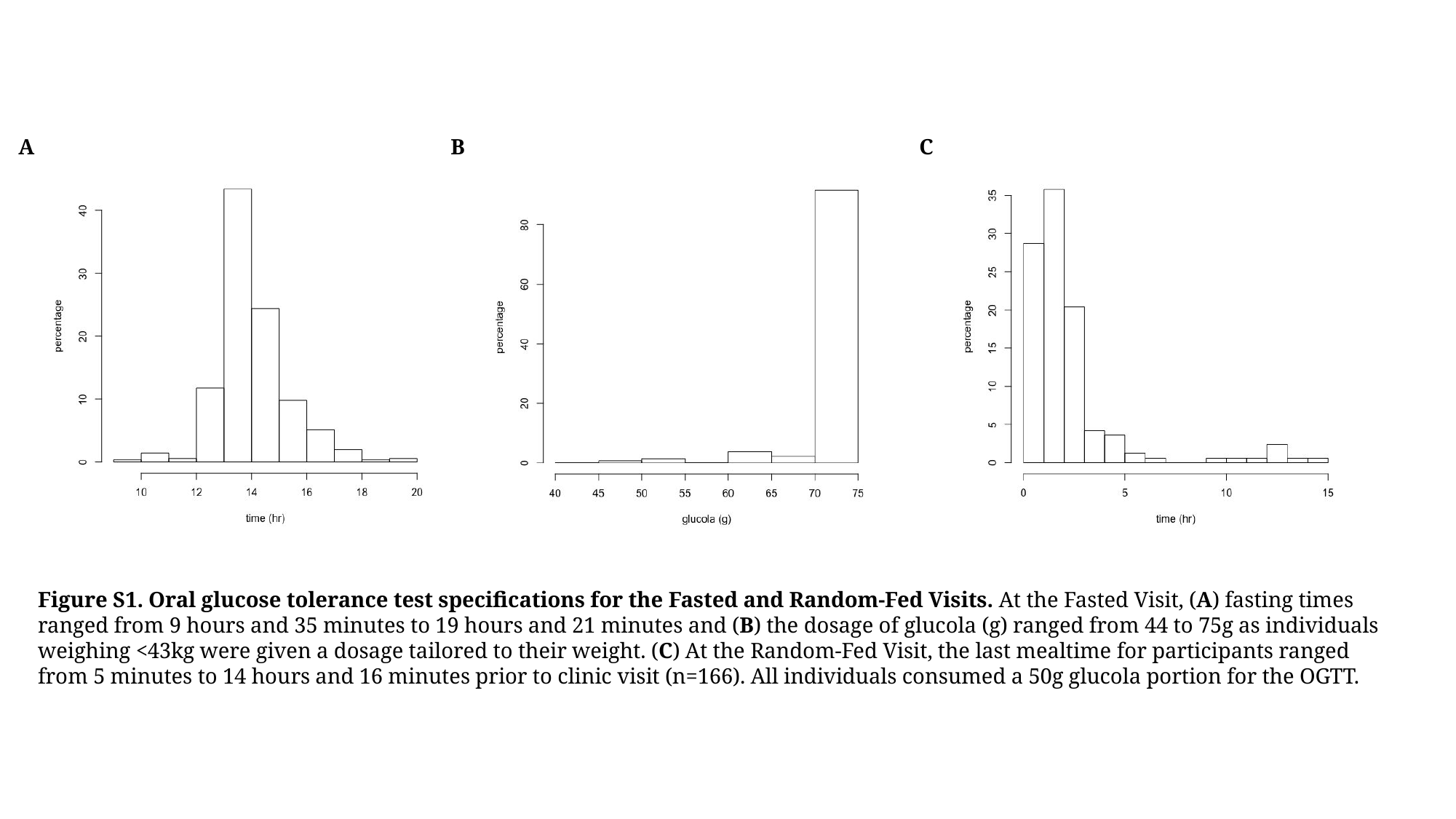

A
B
C
Figure S1. Oral glucose tolerance test specifications for the Fasted and Random-Fed Visits. At the Fasted Visit, (A) fasting times ranged from 9 hours and 35 minutes to 19 hours and 21 minutes and (B) the dosage of glucola (g) ranged from 44 to 75g as individuals weighing <43kg were given a dosage tailored to their weight. (C) At the Random-Fed Visit, the last mealtime for participants ranged from 5 minutes to 14 hours and 16 minutes prior to clinic visit (n=166). All individuals consumed a 50g glucola portion for the OGTT.

## Slide 2
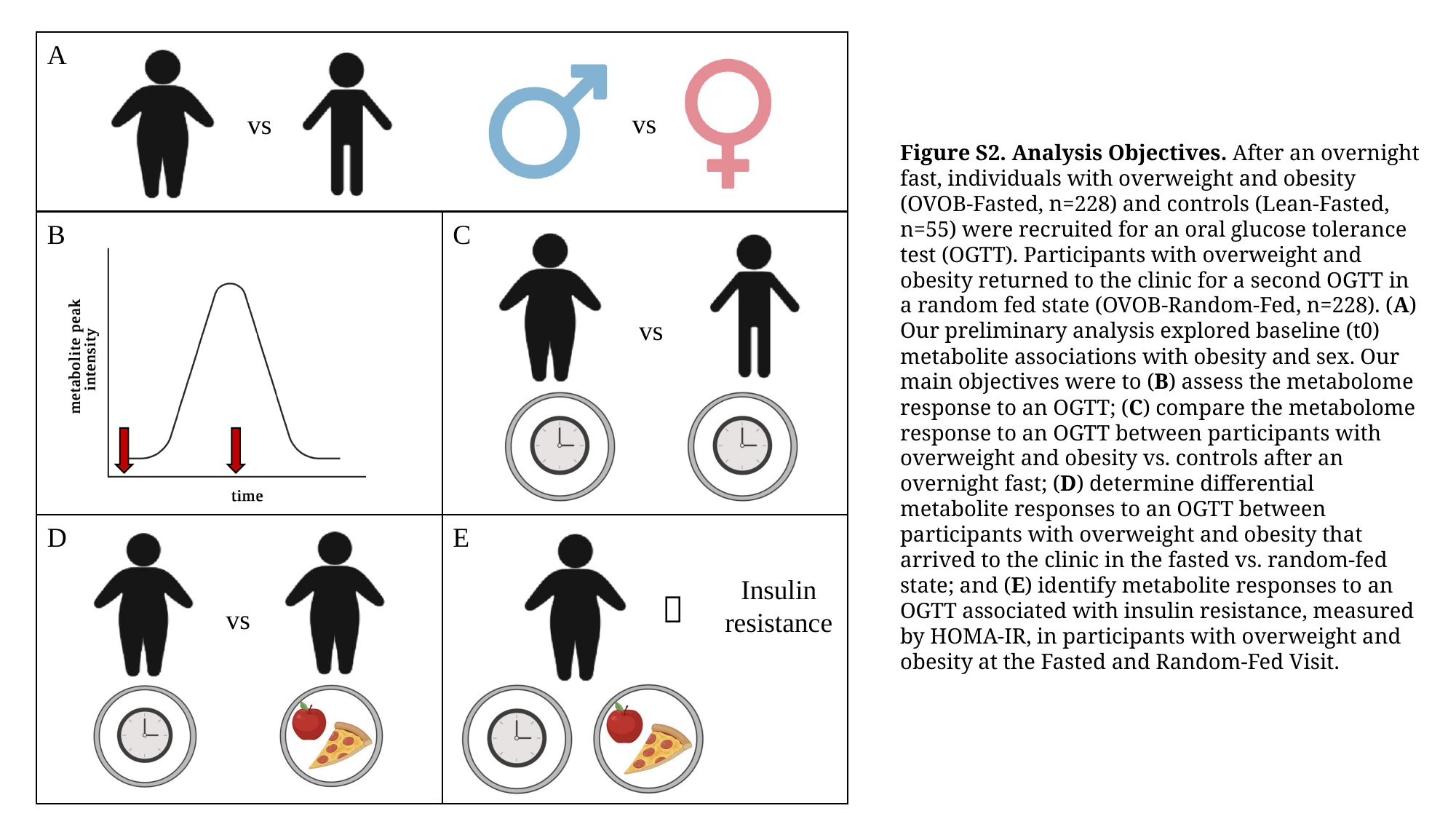

A
vs
vs
Figure S2. Analysis Objectives. After an overnight fast, individuals with overweight and obesity (OVOB-Fasted, n=228) and controls (Lean-Fasted, n=55) were recruited for an oral glucose tolerance test (OGTT). Participants with overweight and obesity returned to the clinic for a second OGTT in a random fed state (OVOB-Random-Fed, n=228). (A) Our preliminary analysis explored baseline (t0) metabolite associations with obesity and sex. Our main objectives were to (B) assess the metabolome response to an OGTT; (C) compare the metabolome response to an OGTT between participants with overweight and obesity vs. controls after an overnight fast; (D) determine differential metabolite responses to an OGTT between participants with overweight and obesity that arrived to the clinic in the fasted vs. random-fed state; and (E) identify metabolite responses to an OGTT associated with insulin resistance, measured by HOMA-IR, in participants with overweight and obesity at the Fasted and Random-Fed Visit.
B
C
vs
D
E
Insulin resistance

vs

## Slide 3
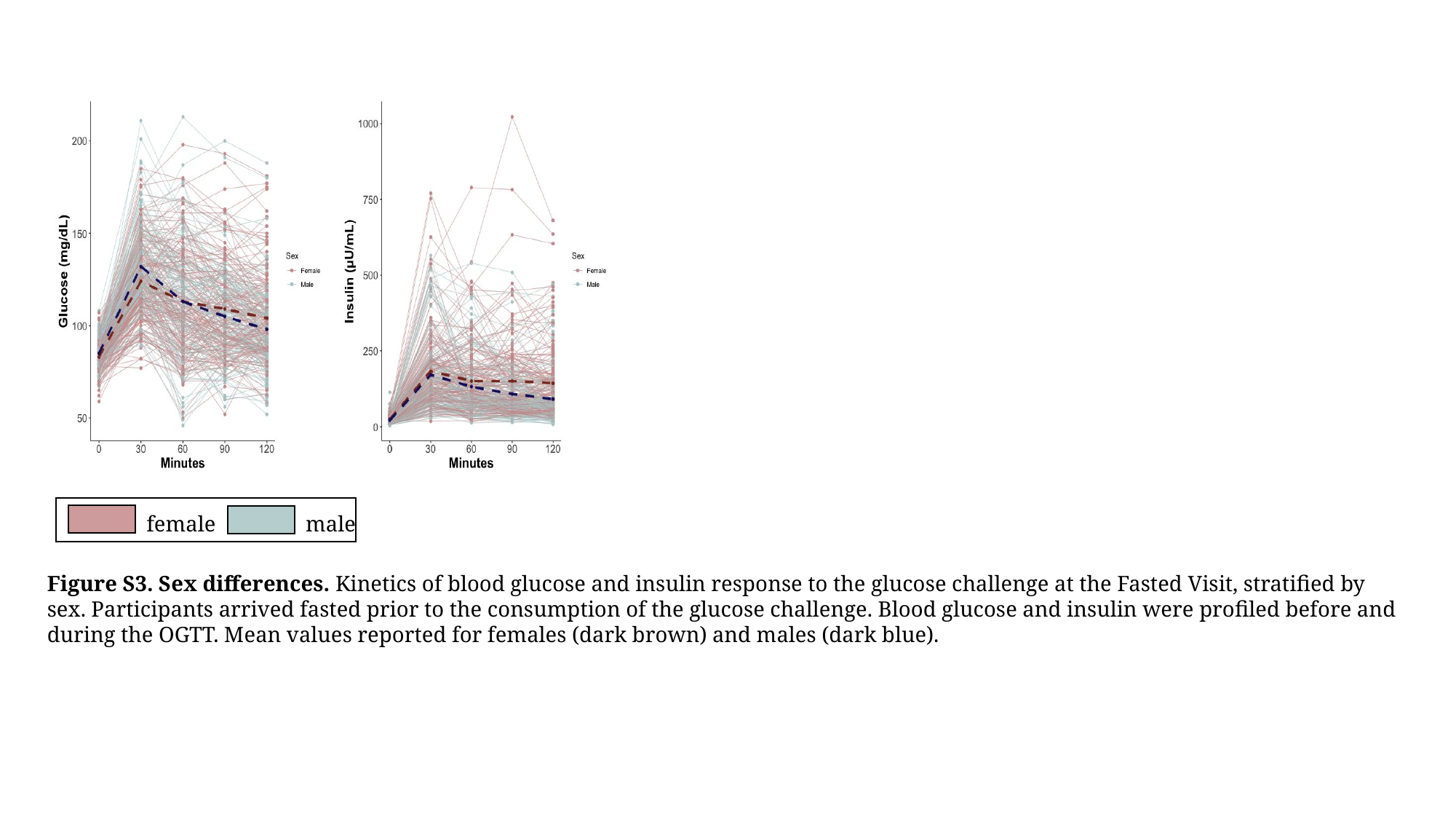

female
male
Figure S3. Sex differences. Kinetics of blood glucose and insulin response to the glucose challenge at the Fasted Visit, stratified by sex. Participants arrived fasted prior to the consumption of the glucose challenge. Blood glucose and insulin were profiled before and during the OGTT. Mean values reported for females (dark brown) and males (dark blue).

## Slide 4
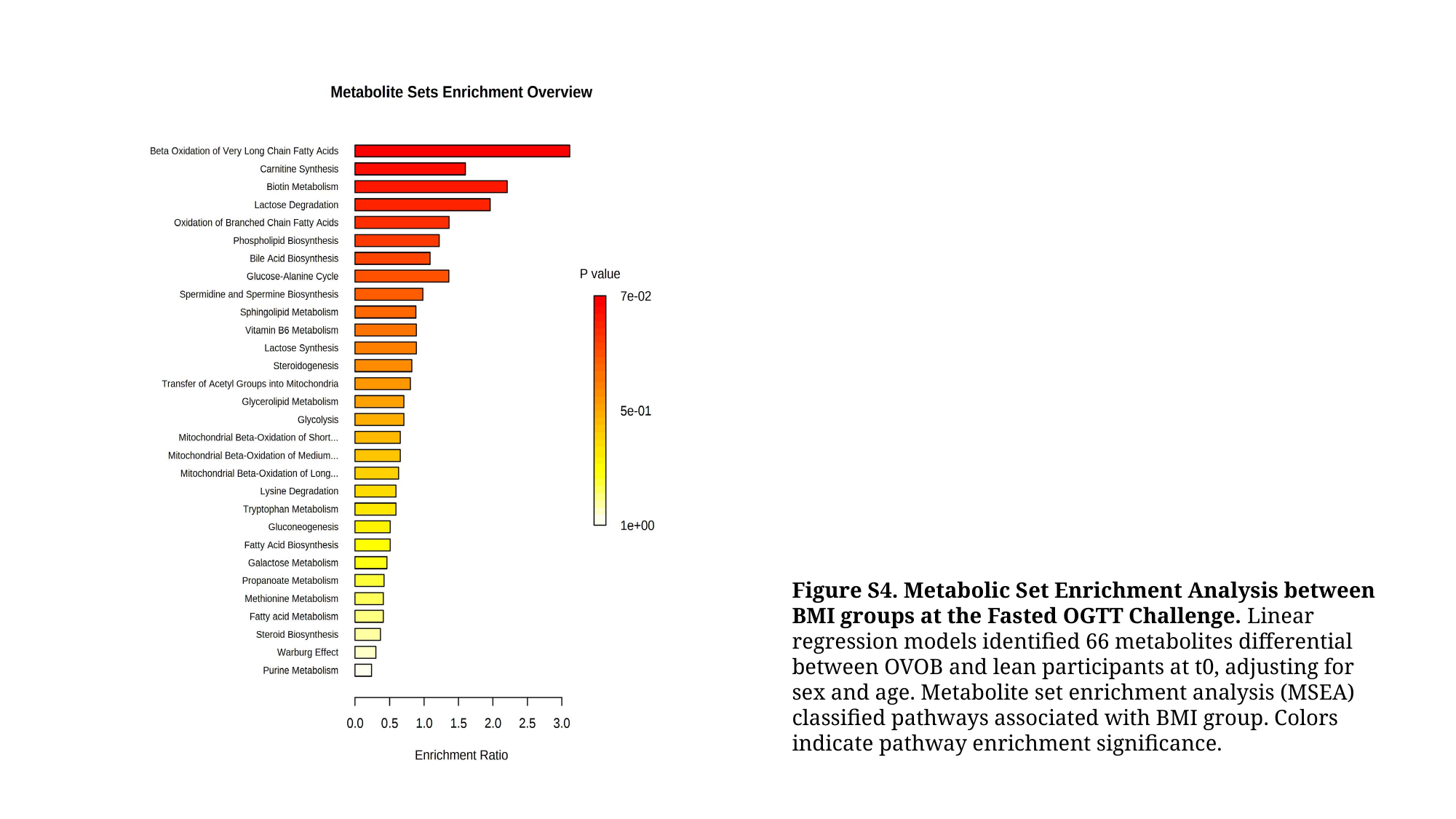

Figure S4. Metabolic Set Enrichment Analysis between BMI groups at the Fasted OGTT Challenge. Linear regression models identified 66 metabolites differential between OVOB and lean participants at t0, adjusting for sex and age. Metabolite set enrichment analysis (MSEA) classified pathways associated with BMI group. Colors indicate pathway enrichment significance.

## Slide 5
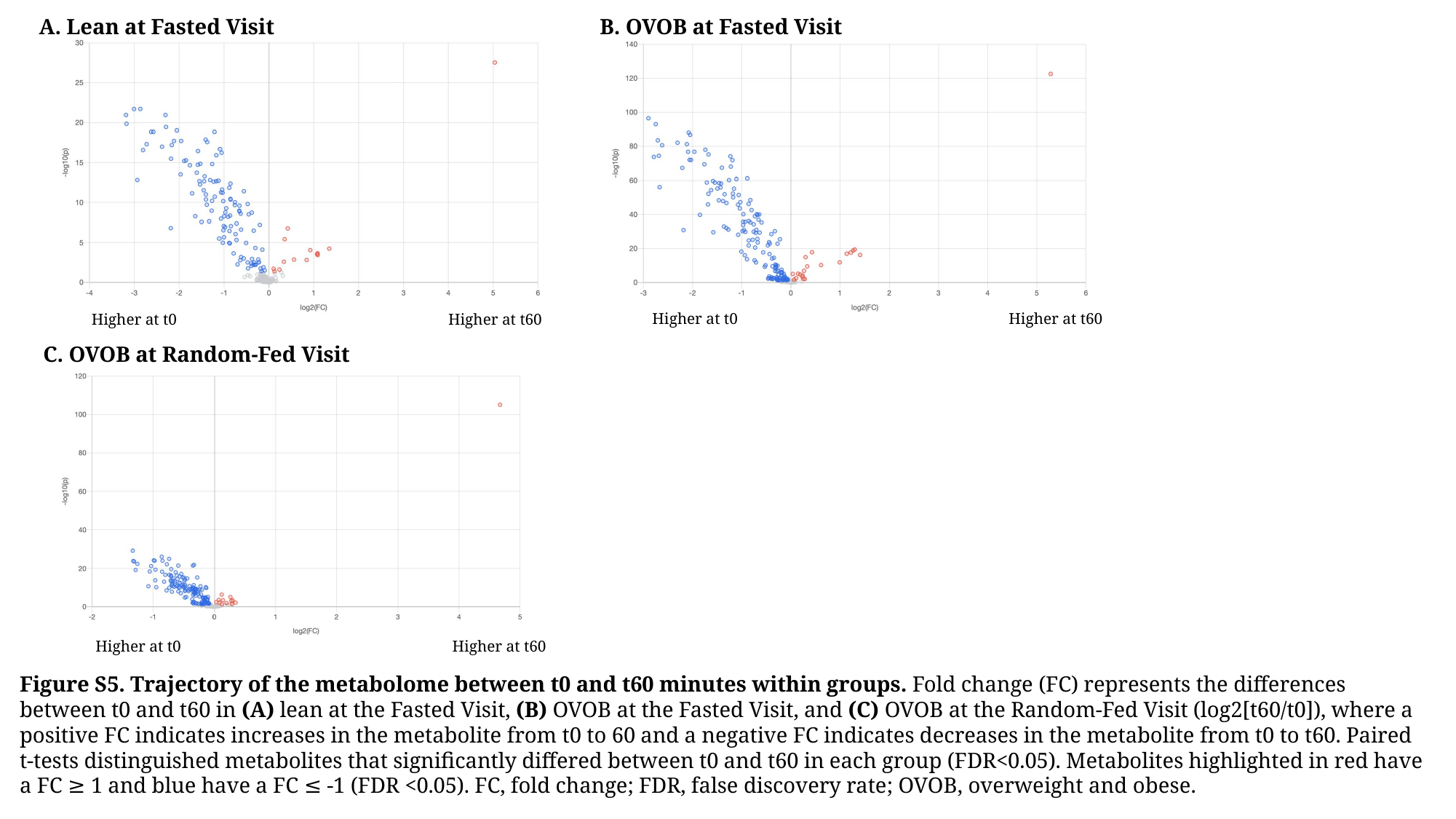

B. OVOB at Fasted Visit
Higher at t0
Higher at t60
A. Lean at Fasted Visit
Higher at t0
Higher at t60
C. OVOB at Random-Fed Visit
Higher at t0
Higher at t60
Figure S5. Trajectory of the metabolome between t0 and t60 minutes within groups. Fold change (FC) represents the differences between t0 and t60 in (A) lean at the Fasted Visit, (B) OVOB at the Fasted Visit, and (C) OVOB at the Random-Fed Visit (log2[t60/t0]), where a positive FC indicates increases in the metabolite from t0 to 60 and a negative FC indicates decreases in the metabolite from t0 to t60. Paired t-tests distinguished metabolites that significantly differed between t0 and t60 in each group (FDR<0.05). Metabolites highlighted in red have a FC ≥ 1 and blue have a FC ≤ -1 (FDR <0.05). FC, fold change; FDR, false discovery rate; OVOB, overweight and obese.

## Slide 6
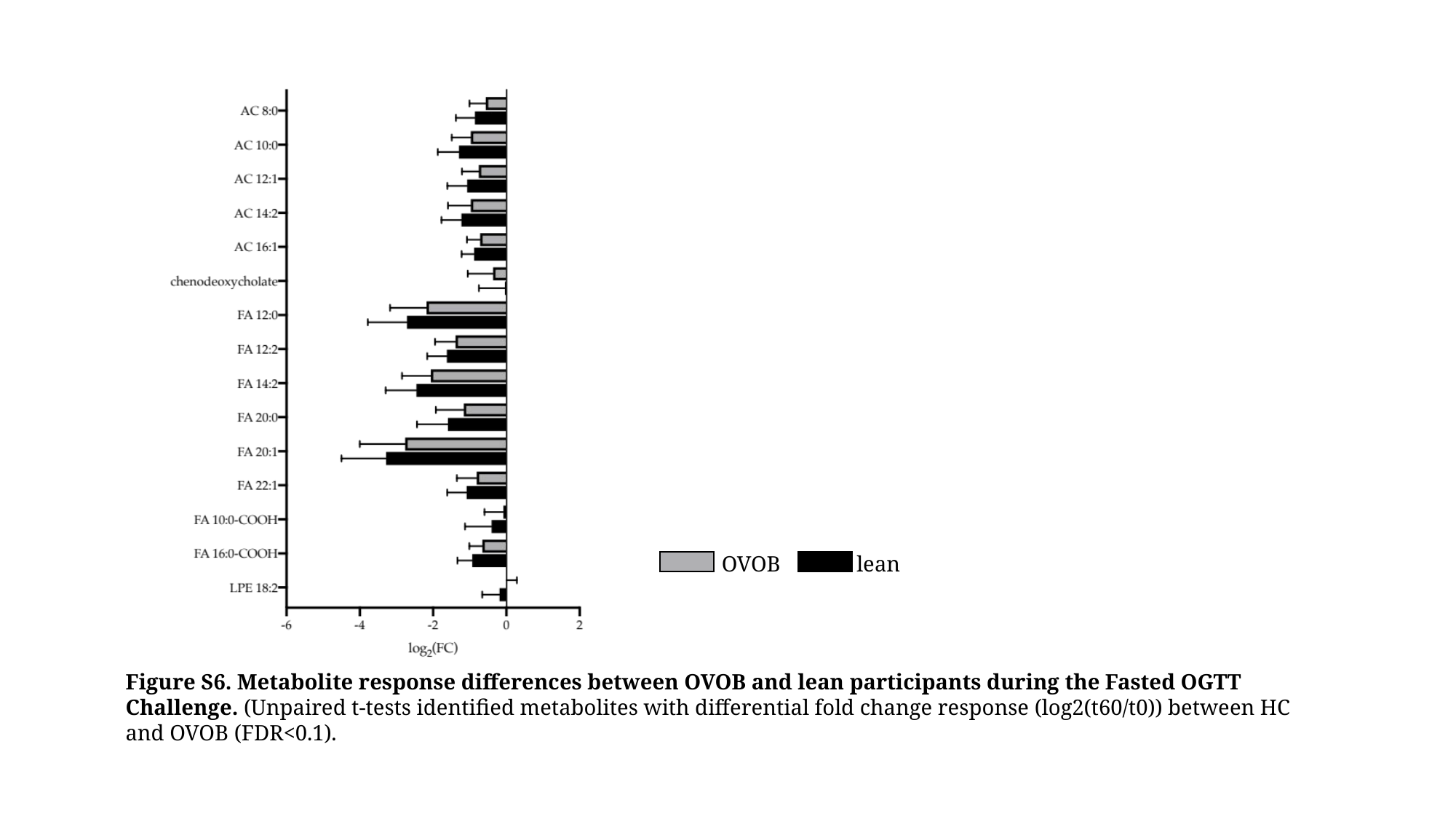

OVOB
lean
Figure S6. Metabolite response differences between OVOB and lean participants during the Fasted OGTT Challenge. (Unpaired t-tests identified metabolites with differential fold change response (log2(t60/t0)) between HC and OVOB (FDR<0.1).

## Slide 7
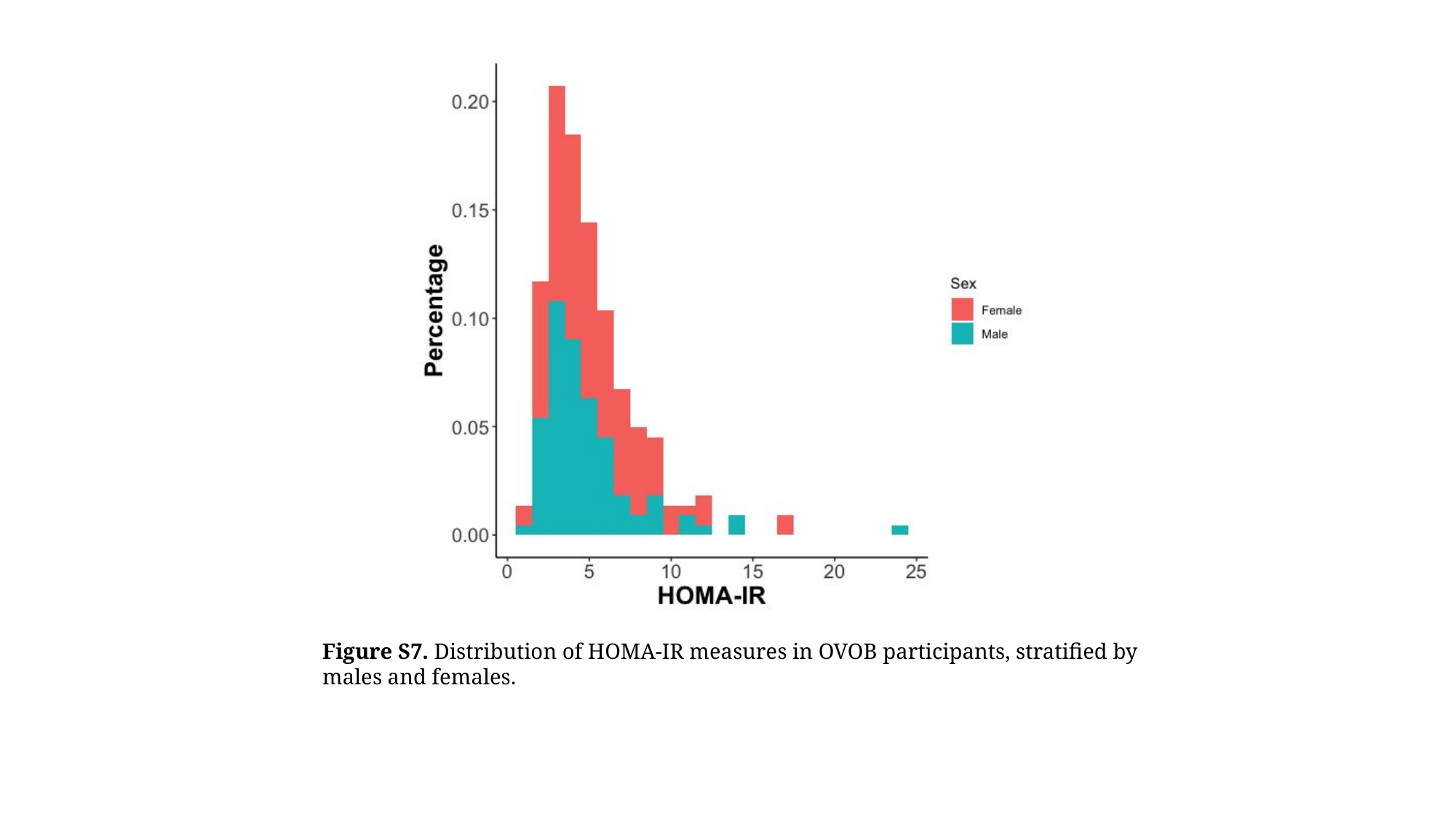

Figure S7. Distribution of HOMA-IR measures in OVOB participants, stratified by males and females.
